# Study protocol to examine the effects of spaceflight and a spaceflight analog on neurocognitive performance: extent, longevity, and neural bases

**DOI:** 10.1186/1471-2377-13-205

**Published:** 2013-12-18

**Authors:** Vincent Koppelmans, Burak Erdeniz, Yiri E De Dios, Scott J Wood, Patricia A Reuter-Lorenz, Igor Kofman, Jacob J Bloomberg, Ajitkumar P Mulavara, Rachael D Seidler

**Affiliations:** 1 School of Kinesiology, University of Michigan, Ann Arbor MI, USA; 2 Wyle Life Sciences, Houston, TX, USA; 3 College of Liberal Arts and Sciences, Pacific Azusa University, Los Angeles, CA, USA; 4 NASA Johnson Space Center, Houston, TX, USA; 5 Department of Psychology, University of Michigan, Ann Arbor, MI, USA; 6 Neuroscience Laboratory, NASA Johnson Space Center, Houston, TX, USA; 7 Universities Space Research Association, Houston, TX, USA; 8 Neuroscience Program, University of Michigan, Ann Arbor, MI, USA; 9 Institute of Gerontology, University of Michigan, Ann Arbor, MI, USA

**Keywords:** Space flight, Astronauts, Microgravity, Sensorimotor feedback, Cognition, Neuroimaging, MRI, Longitudinal studies, Bed rest

## Abstract

**Background:**

Long duration spaceflight (i.e., 22 days or longer) has been associated with changes in sensorimotor systems, resulting in difficulties that astronauts experience with posture control, locomotion, and manual control. The microgravity environment is an important causal factor for spaceflight induced sensorimotor changes. Whether spaceflight also affects other central nervous system functions such as cognition is yet largely unknown, but of importance in consideration of the health and performance of crewmembers both in- and post-flight. We are therefore conducting a controlled prospective longitudinal study to investigate the effects of spaceflight on the extent, longevity and neural bases of sensorimotor and cognitive performance changes. Here we present the protocol of our study.

**Methods/design:**

This study includes three groups (astronauts, bed rest subjects, ground-based control subjects) for which each the design is single group with repeated measures. The effects of spaceflight on the brain will be investigated in astronauts who will be assessed at two time points pre-, at three time points during-, and at four time points following a spaceflight mission of six months. To parse out the effect of microgravity from the overall effects of spaceflight, we investigate the effects of seventy days head-down tilted bed rest. Bed rest subjects will be assessed at two time points before-, two time points during-, and three time points post-bed rest. A third group of ground based controls will be measured at four time points to assess reliability of our measures over time. For all participants and at all time points, except in flight, measures of neurocognitive performance, fine motor control, gait, balance, structural MRI (T1, DTI), task fMRI, and functional connectivity MRI will be obtained. In flight, astronauts will complete some of the tasks that they complete pre- and post flight, including tasks measuring spatial working memory, sensorimotor adaptation, and fine motor performance. Potential changes over time and associations between cognition, motor-behavior, and brain structure and function will be analyzed.

**Discussion:**

This study explores how spaceflight induced brain changes impact functional performance. This understanding could aid in the design of targeted countermeasures to mitigate the negative effects of long-duration spaceflight.

## Background

Over the last two decades, several studies have been published on the impact of long-duration (i.e., 22 days or longer) spaceflight on the central nervous system (CNS). While the effects of spaceflight on the sensorimotor systems and the resulting difficulties that astronauts experience with posture control, locomotion, and manual control are well documented [1-5], it is unclear if spaceflight is associated with cognitive dysfunction [6]. As a result of poor control conditions and inadequate power in the published studies on the neural correlates of spaceflight, a paucity of knowledge exists. In addition, not much is known about the neural mechanisms underlying the behavioral and potential cognitive changes that occur with spaceflight exposure, or their development and recovery over time. In consideration of the health and performance of crewmembers in flight and post-flight, we are conducting a controlled prospective longitudinal study to investigate the effects of spaceflight on the extent, longevity and neural bases of sensorimotor, cognitive, and neural changes.

### Effects of spaceflight on motor behavior and cognitive functioning

#### Motor behavior

While in space, the visual, vestibular and proprioceptive systems of astronauts adapt to microgravity [5,7,8]. Following their return to earth, astronauts have to readapt to Earth’s gravity. During this re-adaptation period disturbances have been reported in spatial orientation, posture, gait, and eye-hand coordination [5,9], which can potentially be ascribed to the central reinterpretation of multiple sensory inputs [8].

Studies conducted at National Aeronautics and Space Administration (NASA) Johnson Space Center’s Neuroscience Laboratories on the effects of spaceflight on human motor behavior have reported post-flight changes in locomotor control and body segmental coordination. Studies including subjects that completed missions with a duration of up to two weeks showed alterations in muscle activation variability [10], increased variability in ankle and knee joint motion [11], alterations in head-trunk coordination and reduced visual acuity during walking [12], impairment in the ability to coordinate effective landing strategies during jump tasks [13], with poorer balance and problems with postural stability lasting up to ten days post-flight [5,8,14,15]. In addition to alterations in muscle activation variability [16-18] that were observed after short duration missions, disruptions in lower limb kinematics leading to reduced toe clearance [19], and poorer ability to complete challenging locomotor maneuvers [20] were observed in subjects after long-duration spaceflight missions (i.e. with a duration of three to six months).

Most of the post-flight vestibulo-motor disturbances have been attributed to reorganization of information from the otoliths specifically, which signal orientation of the head with respect to a gravitational reference vector [21]. However, it is clear that behaviors mediated by intravestibular interaction of the semicircular canals and otoliths are affected by microgravity exposure as well [22]. Other proposed mechanisms for the association between microgravity and reduced motor control abilities include changes in bodily fluid shifts that result in disruption of thalamic function [23], and direct effects on the ocular and visual neural systems [24]. Moreover there may be neuroplasticity occurring in association with adaptive behavioral modifications as astronauts learn to control their movements in the microgravity environment. These adaptations may be the result of restitution and/or substitution of control processes. Restitution refers to functional recovery through physiological recovery as a result of spontaneous tissue heal and neural pathway re-activation. Substitution is the functional restoration via system reorganization, where certain brain areas are recruited to take over the function of previously relied upon brain regions, or compensation which refers to adaptation on the functional and neural levels [25]. Which of these principles are responsible or play the bigger role in astronaut’s adaptation to the microgravity environment is yet to be discovered.

#### Cognition

Although studies have reported little to no change in overt cognitive abilities during flight and post-flight while using available monitoring technology, the few studies that have investigated the effects of spaceflight on cognition in humans reported that dual-tasking of cognitive and motor behaviors is significantly impaired during the initial period of adaptation to a microgravity environment [26-28] and over a longer six month mission [2]. Furthermore, they indicated that the increased demands of motor control in space can interfere with simultaneous cognitive task performance. However, results from a recently published review emphasized that due to inadequate study designs it remains unclear whether and how spaceflight is associated with cognitive dysfunction [6].

### Central nervous system plasticity

Whether and to what extent the reported sensorimotor and potential cognitive changes are related to spaceflight-induced brain structural changes, apart from vestibular reorganization, or more peripheral changes such as muscle unloading and bodily fluid shifts, is not yet known. However, existing evidence supports that the brain may undergo structural and functional remodeling similar to that seen in bone and muscle, as a result of the interaction between exposure to microgravity, vascular changes, and radiation associated with spaceflight. Plasticity may occur, including cortical reorganization associated with sensorimotor adaptation, compensatory processing, and brain volumetric alterations [14,29-32].

#### Microgravity

To the best of our knowledge, no studies have examined the potential effects of microgravity on brain structure in humans. However, animal research demonstrates structural brain changes after microgravity exposure. These effects were most notable in the somatosensory cortex [29,30,33,34] and the cerebellum [29] and include a decreased number of synapses and the degeneration of axonal terminals. Ross (1993, 1994) has demonstrated that hair cells in the rat utricular macula, the part of the vestibular system that perceives changes in longitudinal acceleration and gravity, undergoes extensive plasticity as a result of spaceflight, with a 40% to 55% increase in synapse number [31,32]. This plasticity remained evident following flight, even after posture control in the rats had returned to normal, suggestive of compensatory post flight control processes. Moreover, these results were observed in animals after one [29], nine [31,32], and fourteen days [33] in a microgravity environment.

#### Indirect effects of microgravity: vascular changes

Changes in cerebral blood flow as a result of microgravity exposure may also contribute to brain reorganization. Following spaceflight, astronauts have reduced arterial pressure and cerebral blood flow velocity as measured with transcranial Doppler (i.e. ultrasound) [35]. Similarly, Gazenko et al. [36] found that astronauts show reduced cerebral blood flow pulsatility, as measured with impedance rheography (a method used to study the filling of a part of the body with blood by graphically recording the fluctuations in the resistance of that part of the body), when in a head-down tilt posture following spaceflight. Other studies have demonstrated a microgravity dose-dependent effect, with cerebral vasoconstriction following long-term flight not resolved after a period of five weeks [36,37]. It is hypothesized that this increased vasoconstriction is an adaptive response to the increased cranial pressure experienced while in the microgravity environment. Blood vessel remodeling can occur relatively quickly, with as little as two weeks of head-down tilt on Earth resulting in increased vessel wall thickness and vessel diameter in the brain vasculature and concomitant decreases in the lower extremity vasculature [38,39].

#### Galactic cosmic radiation

The heavy ion component of cosmic radiation is potentially harmful for the CNS [40]. Studies have shown that exposing mice to a space radiation analog by directly irradiating them (100/150 cGy; irradiation duration ~2 min) adversely affects their memory and recognition, weeks to months post-exposure [41,42]. In addition, radiation exposure has been associated with acceleration of amyloid-β plaque pathology [41], which in itself is associated with an increased risk of Alzheimer’s disease in humans [43]. Nevertheless, the effects of cosmic radiation on the CNS are generally of greater concern for long-duration exploration missions (e.g. to the moon or Mars), and are less likely to result from short missions in low Earth orbit, where the Earth’s magnetic field is still protective against galactic cosmic rays, and in which the composition of these rays is less hazardous than beyond low Earth orbit [44].

#### Other risk factors

Other risk factors that may affect brain function that astronauts encounter, that are unrelated to the extraterrestrial environment, include stress, scarcity of resources [6], sleep loss, fatigue, circadian desynchronization, and work overload [12]. These can potentially be modeled with spaceflight analogs such as wintering over at Arctic field stations.

#### Multitude of risk factors

**Figure 1:**
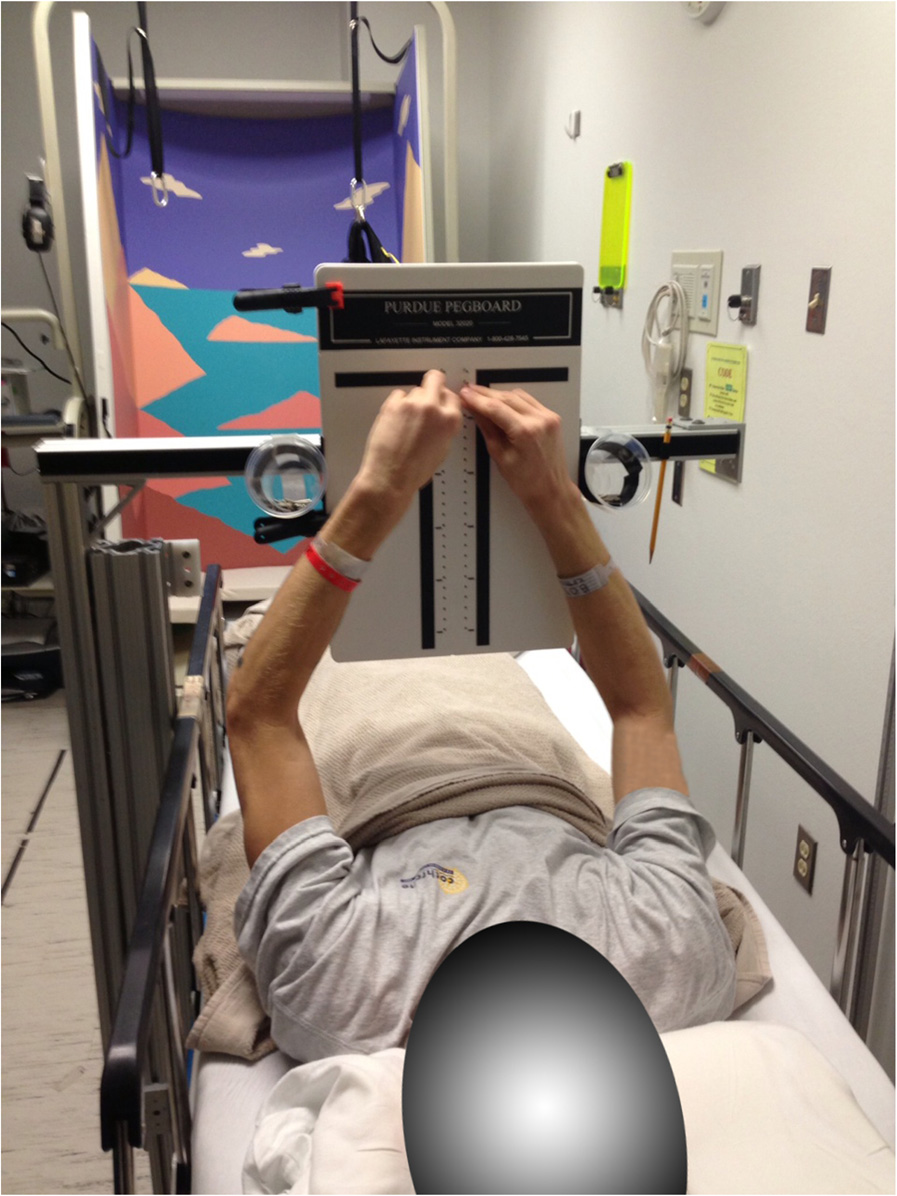
Bed rest subject performing the Purdue Pegboard task.

Determining the mechanisms behind the effects of spaceflight on the brain requires distinguishing the contribution of factors that are unique to the extraterrestrial environment from other characteristics of spaceflight. Currently, one of the risk factors that is expected to have a relatively large impact on brain structure and function during and after long duration spaceflight is the microgravity environment. To parse out the effect of microgravity from the overall effects of spaceflight, bed rest analog studies have been designed [45]–[47]. In these studies, participants are required to stay in bed with their heads tilted 6° below their feet for a number of consecutive days (see Figure 1). Under these conditions, the effects of microgravity such as unloading, reduced sensory inputs, and increased cephalic fluid distribution can be studied apart from other spaceflight effects on the brain. At this point, only two studies have investigated the effect of bed rest on cognitive functioning [48,49]. These studies have not revealed significant adverse cognitive effects of bed rest. However, their sample sizes were small (n = < 15) and cognitive functioning was measured with an extended cognitive screening instrument (i.e. WinSCAT, [50]), rather than with a thorough battery of neuropsychological tests. In addition, these studies have been conducted in healthy subjects that are generally able to compensate for small to moderate cognitive problems to a certain extent [51,52]. Up until now, only one study has investigated the effect of microgravity on brain activation during rest. This study by Liao et al. (2012) revealed a change in resting state connectivity of the left thalamus with a variety of brain cortical regions after a period of 72 hours of head down tilt bed rest [23]. This altered activation may potentially contribute to reduced motor control abilities in astronauts in a microgravity environment. Currently there are no studies that have investigated the association between bed rest, cognitive functioning, sensorimotor control, and brain function and structure.

### Study rationale and clinical relevance

Given the lack of data in humans, there is a fundamental gap in understanding the extent, longevity, and neural bases of long-duration spaceflight-induced changes in neurocognitive performance. Changes in brain structure and function may play a direct role in crew performance and thus operational success. Furthermore, it may impact the long-term health of astronauts, particularly in advanced age when the potential effects of spaceflight may interact with brain volume loss and functional reorganization that occurs with normal human aging [53-55]. NASA crewmembers may be at risk of accelerated aging effects if substantial volumetric degeneration and functional reorganization in the brain occurs during spaceflight. With the current study, we hope to identify the underlying neural mechanisms and operational risks of spaceflight-induced changes in behavior, and to identify whether a return to normative behavioral function following re-adaptation to Earth’s gravitational environment is associated with a restitution of brain structure and function or instead is supported by substitution with compensatory brain processes. Identification of neurocognitive changes occurring as a function of spaceflight is the first step towards development of targeted countermeasures. These measures could be successful at slowing, and in some cases even reversing any potential spaceflight associated brain changes, in a similar way to those that have been developed to counteract the effects of normal aging [56-59].

Cognitive and behavioral measures will be collected from astronauts before, during, and after spaceflight. Functional and structural brain scans will be obtained at multiple time points pre- and post-flight. A group of individuals that are participating in an ongoing head down tilt bed rest study, and a group of ground-based control subjects, will also complete the exact same assessments.

This is the first prospective longitudinal study assessing both spaceflight induced functional and structural brain changes and their relationship to sensorimotor and cognitive functioning.

### Objectives

The aim of our studies are to a) identify changes in brain structure and function that occur with spaceflight and prolonged bed rest and characterize their recovery time course; b) assess whether and how these changes impact behavioral and neurocognitive performance; and c) parcel out the contribution of microgravity to the potential brain functional and structural changes.

### Hypotheses

We hypothesize that measures of brain structure, function, and network integrity will change from pre to post flight in crewmembers, and to a lesser extent in bed rest participants with accumulating time, but not in control subjects. Moreover, we predict that these changes will correlate with indices of cognitive, sensory, and motor function in a neuroanatomically selective fashion.

## Design/methods

To investigate the effects of long-duration spaceflight on brain function and structure, we will perform an extensive neuro-imaging protocol, including task-based BOLD (blood oxygenation level-dependent) Magnetic Resonance Imaging (MRI), resting state functional connectivity MRI, high-resolution structural MRI, and diffusion weighted MRI. Potential demographic confounders will be collected for all subjects.

### Design

The current study includes three groups for which each the design is single group with repeated measures. Thus, astronauts and bed rest study subjects will serve as their own controls from pre flight to in flight and post flight test points, and pre bed rest to in bed rest and post bed rest respectively (see Table 1). In addition, the bed rest study will serve as an experimental analog for spaceflight because extended exposure to a head-down tilt position can duplicate many of the effects of a low-gravity environment [60]; thus, the study will consist of both within subject and between subjects comparisons.

**Table 1:** Testing timeline for astronauts and bed rest subjects

**Astronauts**	**Pre launch**					**Flight day**						**Return**	
**Day:**	**-180**	**-90**				**30**	**90**	**150**				**+90**	**+180**
**Bed rest subjects**	**Pre bed rest**		**In bed rest**						**Post bed rest**				
**Day:**	**-12**	**-7**	**7**	**30**	**65-70**				**+0**	**+8**	**+12**		
**Behavioral measures**													
Thurston's card rotation task	X	X	X	X	X					X	X	X	X
Cube mental rotation task	X	X	X	X	X	X	X	X		X	X	X	X
Rod and frame test	X	X	X	X	X					X	X	X	X
Digit symbol substitution task of the WAIS	X	X	X	X	X					X	X	X	X
Purdue pegboard test	X	X	X	X	X					X	X	X	X
Computerized dynamic posturography	X	X							X	X	X	X	X
Functional mobility test	X	X							X	X	X	X	X
**Structural neuro-imaging**													
High resolution T1‒weighted imaging	X	X	X	X	X					X	X	X	X
Diffusion tensor imaging	X	X	X	X	X					X	X	X	X
**Functional neuro-imaging**													
Functional connectivity MRI	X	X	X	X	X				X	X	X	X	X
VEMP	X	X	X	X	X				X	X	X	X	X
Dual task	X	X	X	X	X	•	•	•	X	X	X	X	X
Sensorimotor adaptation task	X	X	X	X	X	•	•	•	X	X	X	X	X
Spatial working memory task	X	X	X	X	X				X	X	X	X	X
Foot tapping	X	X	X	X	X				X	X	X	X	X

WAIS = Wechsler adult intelligence scale; MRI = Magnetic resonance imaging; VEMP = Vestibular evoked myogenic potential; ● = fMRI paradigms of which the behavioral measure is completed while in space.

Comparing longitudinal outcome measures (i.e. brain structure and function) in astronauts with those in bed rest subjects will provide insight into the mechanisms behind the potential effects of spaceflight on the brain.

To evaluate the stability and reliability of our behavioral and MRI measures over time, we will also run a parallel study with ground-based control participants testing across multiple time points.

#### Testing timeline

Astronauts will be assessed at nine time points: two assessments will take place before launch (approximately 180 days and 90 days before launch), three assessments will take place while in flight (approximately day 30, 90, and 150); and four assessments will take place post flight (approximately 1-4, 30, 90 and 180 days post flight) (see Table 1).

Bed rest subjects will remain in bed with their heads tilted down for 70 consecutive days. Behavioral measures and neuroimaging assessments will be obtained at seven time points: a) two measurements will take place approximately 12 and 7 days before bed rest; b) two measurements will take place at approximately 7 and 30 days in bed rest; and c) three measurements post bed rest: one on 65 days into a 70 day campaign as close to day 0 when the subject gets up from bed (for all measures except those requiring upright stance), day 0 of standing up from bed for tests requiring upright stance, day 8 and 12 after the subject stands up for all tests (see Table 1).

Ground-based control subjects will be completing the exact same measures as the astronauts at four time points, with a one-month interval between each consecutive pair of time points.

### Participants

#### Recruitment

##### Astronauts

Thirteen astronauts will be recruited from crewmembers designated to visit the International Space Station (ISS). All astronauts who are assigned to a mission are given an opportunity to participate in a number of studies. Astronaut subjects who agree to participate in the study and whose in- and post- flight schedules match the study time line requirements are recruited into the study.

#### Bed rest subjects

The bed rest program is a framework designed by NASA that offers researchers from various backgrounds the possibility to study bed rest as an experimental analog for space flight because extended exposure to a head-down tilt position can duplicate many of the effects of a low-gravity environment [48,60]. Participants will be recruited through the bed rest facility located at the University of Texas Medical Branch (Galveston, TX) and will participate in several studies as long as they are not interfering with each other.

Bed rest subjects are aged 18-60 years and will be required to pass an Air Force Class III equivalent physical examination. Female subjects in this study will model those in the astronaut population for whom participation in space missions is not allowed during pregnancy. Therefore, for female subjects, a non‒positive result from a pregnancy test will be required prior to inclusion in the study and prior to each experimental session.

#### Ground-based control subjects

Thirteen control subjects will be recruited from the Test Subject Facility at the NASA Johnson Space Center. These subjects need to be age matched with our astronaut subjects and will be required to pass an Air Force Class III equivalent physical examination.

#### Sample size calculation

To calculate the sample size for this study we used data from the Functional Mobility Test (FMT). The FMT was designed to evaluate an astronaut’s ability to complete challenging locomotor maneuvers similar to those encountered during an egress from a space vehicle following long-duration space flight [20]. To perform the FMT subjects walked at a self-selected pace through an obstacle course set up on a base of medium density foam. The foam provided an unstable surface that increased the challenge of the test. The 6.0 m × 4.0 m course consisted of several pylons and obstacles made of foam. Subjects were instructed to walk through the course as fast as possible without touching any of the objects on the course. FMT data were used to calculate the sample size as it is the only pre/post flight data currently available with the longest recovery times (~15 days). If the other tests that are used in this study are similar to the FMT in sensitivity to spaceflight, we would fully expect to reject H_0_ for all tests. For example, from FMT results on 18 long-duration international space station (ISS) subjects, we found the mean change in log transit times to be 1.68 log sec with a standard deviation of 0.60 log sec [20]. With such a large signal-to-noise ratio and normally distributed differences the power of the t-test against H_0_ is virtually 1.0, even with as few as 10 subjects. Even if an outcome has only half the sensitivity of the FMT to spaceflight, the power with 10 subjects would still be 0.975. However, it is also important to have enough subjects to accurately estimate the mean change. If the sensitivity of a test to spaceflight were similar to that of the FMT, it would take about 13 subjects to produce a coefficient of variation of 10% for the estimated mean change post flight with respect to preflight performance. Therefore, under the assumption that sensitivities are comparable, we will require 13 long-duration astronaut subjects. We plan to target 15 subjects so we have a reserve of 2 subjects to account for subject attrition.

By including at least as much bed rest participants and control group participants as astronauts, we will ensure enough power to detect potential changes over time for these populations too.

#### Informed consent

Written informed consent will be obtained from all participants. The current studies were approved by the institutional review boards of the University of Michigan, the University of Texas - Medical Branch (UTMB), and NASA. All studies are being conducted in accordance with the declaration of Helsinki.

#### Remuneration

The astronauts will not receive remuneration for their participation. Bed rest subjects will receive $10 per hour for their participation in addition to a lump sum they receive for participating in the bed rest study. Control subjects will receive $10 per hour for their participation.

## Methods

### Behavioral assessment

Table 2 gives an overview of the cognitive behavioral measures. In general all flight and normative subjects will perform the following behavioral tests while seated in a chair with additional constraints as noted in each tests. For the bed rest and normative subjects the following behavioral tests are performed while lying down in bed supine or on their sides with their superior-inferior axis parallel to the ground.

**Table 2:** Cognitive behavioral measures

**Neuropsychological test**	**Functional area assessed**	**Outcome measure**	**Range**	**Trial (s)**	**Maximum time per trial (seconds)**
Thurston’s card rotation task [61]	Spatial working memory	Number correct^a^	1-20	1	180
Cube mental rotation task [62]	Spatial working memory	a) Number correct^a^	1-26	26	10
		b) Response time (seconds)^b^	0-10		
Rod and frame test [63]	Field perception & dependency	Absolute deviation from vertical (degrees)^b^	0-18	8	n/a
Digit symbol substitution task of the WAIS [64]	Processing speed	a) Number of correctly substituted letters^a^	0-140	1	n/a
		b) Time to complete (seconds)^b^	n/a		
Purdue pegboard test [65]	Dexterity and bimanual coordination	Time needed to put 25 pairs of pins in the board (seconds)^b^	n/a	1	n/a

WAIS = Wechsler adult intelligence scale; ^a^higher score indicates better performance; ^b^less indicates a better performance.

*Spatial working memory* will be assessed with **a**) Thurston’s card rotation task [61]: each item gives a drawing of a card cut into an irregular shape. To its right are six other drawings of the same card, sometimes merely rotated and sometimes turned over to its other side. The subject indicates whether or not the card has been turned over; and **b**) cube mental rotation task [62]: Similar to a), except that target and test shapes are depicted as an assemblage of three-dimensional cubes. These cubes are modeled on those initially developed by Shepherd & Metzler. A three dimensional shape is presented on a computer screen for 3 seconds, followed by a blank screen for 2 seconds. After the delay, two 3D cube images will appear on the screen, of which one will be matching and the other will not be a match of the previous shape. The new shape will be rotated in roll, pitch and yaw with respect to the previous shape. Subjects will be asked to imagine turning of the new image to determine which of the two is a rotated version of the prior object. Subjects will respond by pressing the appropriate key on a gamepad provided them (left or right arrow for the match). The task will consist of 26 trials. Accuracy and response time will be recorded for both tests a and b.

**Figure 2:**
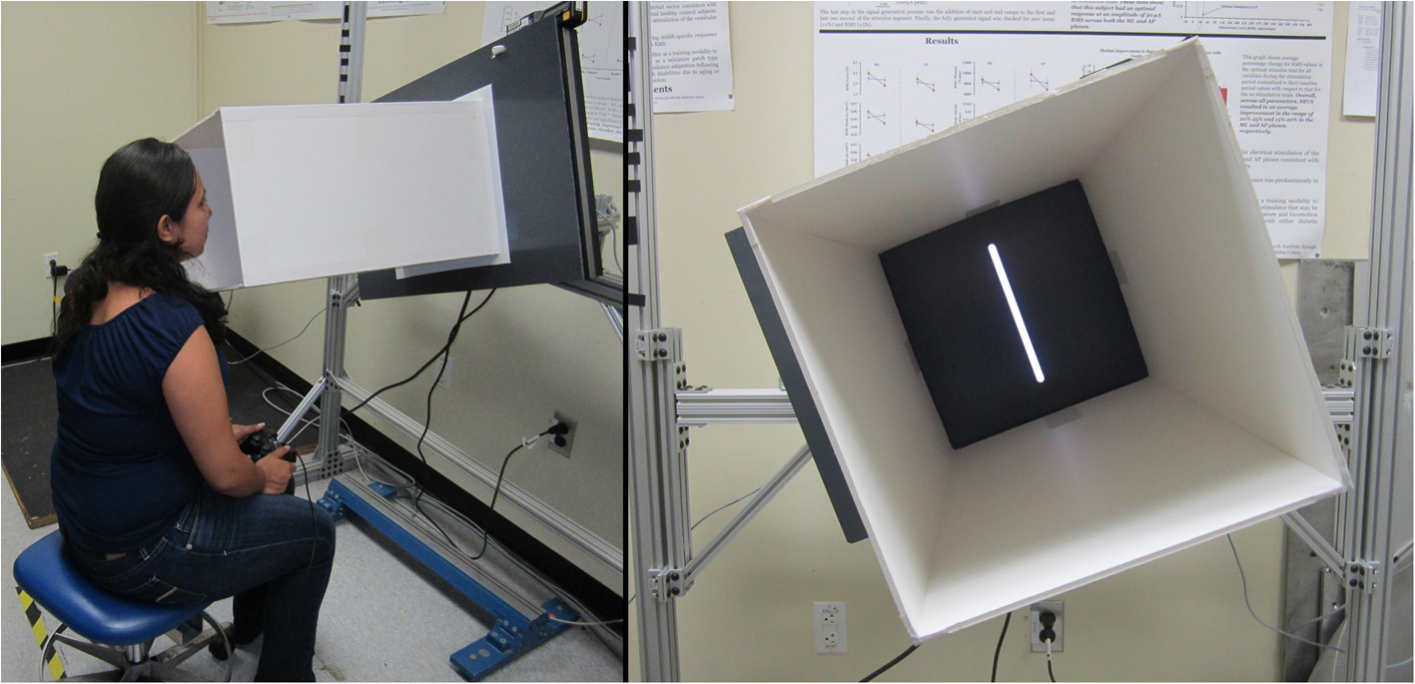
Rod and Frame test.

*Field perception and field dependency* will be measured with the Rod and Frame Test (RFT; see Figure 2) [63]. The RFT is used to determine whether an individual is more or less visually dependent. The RFT consists of a screen where a rod is viewed in a number of different degrees of tilt. A tunnel-like frame (0.6 m) that surrounds the rod is projected towards the subject. Both the degree of tilt of the tunnel frame and the degree of tilt of the rod can be altered. The subject sits in the dark, with the tunnel framing their face, thus removing all earth-fixed visual cues. The subject is then required to align the rod to the upright vertical position by the use of a manual controller. A subject able to successfully align the rod to upright is said to be visually independent. Conversely, if a subject aligns the rod with a tilt bias in the direction of the tilted frame they are classified as visually dependent. The test will be repeated for up to eight trials for two random starting tilt positions of the frame and or the rod (±18°). Proprioceptive cues from the plantar sole contacts with the ground will be limited to the heels by asking subjects to extend their legs during seated trials. The subjects will be scored in degrees of deviation from vertical [63].

*Processing speed* is measured with the digit symbol substitution task of the Wechsler Adult Intelligence Scale-Revised (WAIS-R) [64]: For this paper and pencil test subjects have to match digits with symbols. A key of nine digit-symbol pairs is printed on top of the page. Subjects have to complete the test as fast as possible by writing down the corresponding symbol for each of 140 digits on the page. The score is the time taken to complete the task and the number of correctly matched digits.

*Bimanual coordination* is measured with the Purdue Pegboard test [65]: The test requires participants to manipulate small pegs using both hands simultaneously to put pegs into parallel rows of holes. We measure the time taken to complete all 25 pairs on the board.

**Figure 3:**
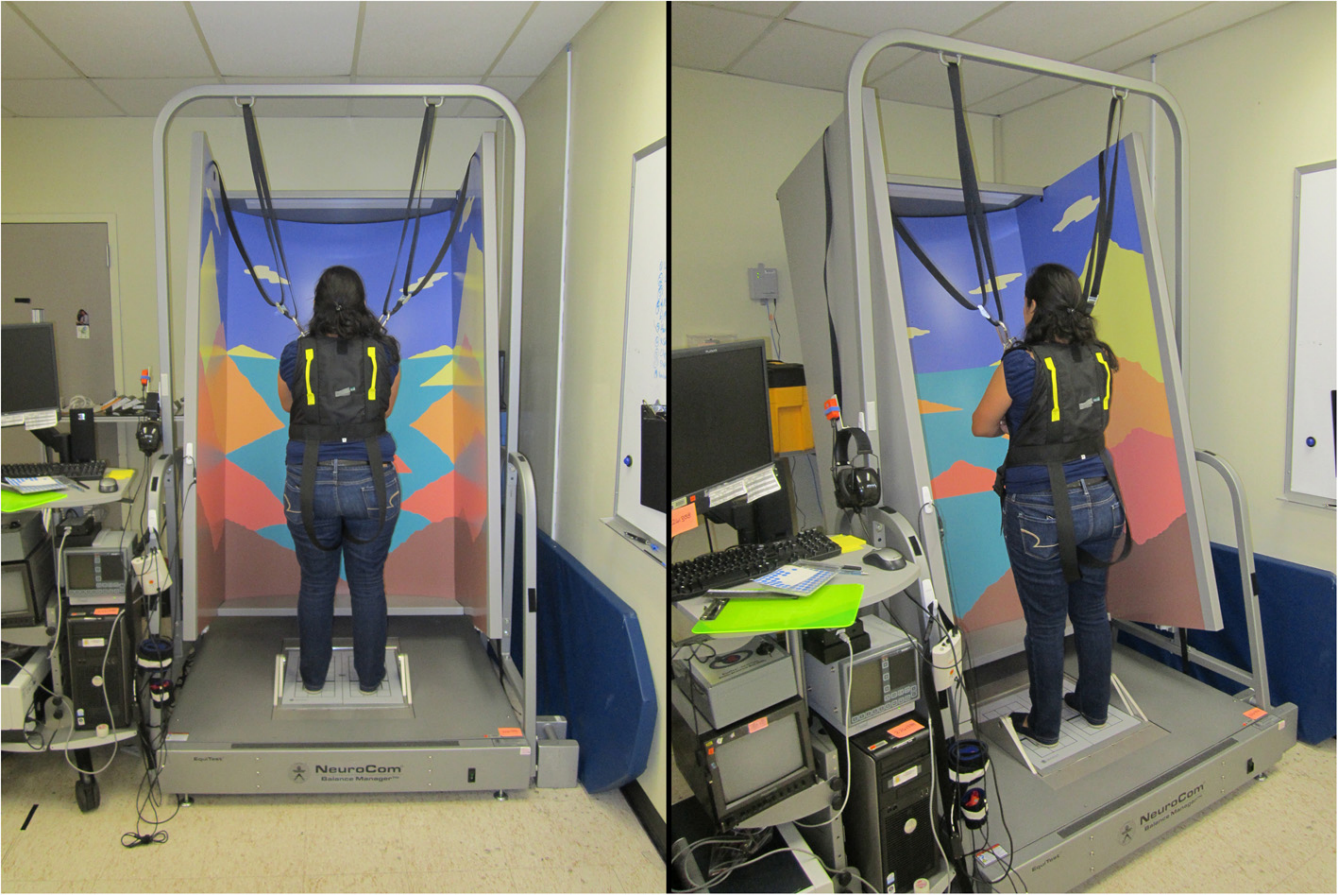
Computerized Dynamic Posturography.

**Figure 4:**
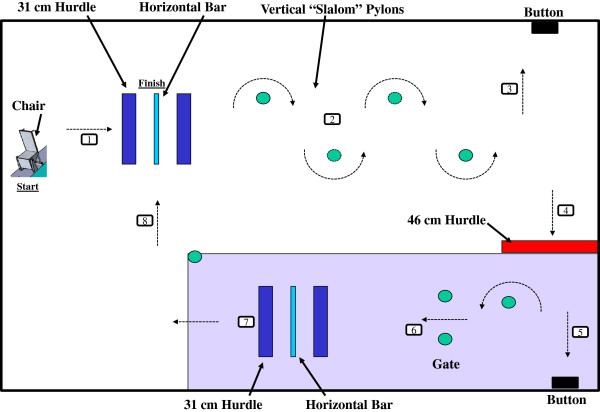
The Functional Mobility Test.

*Posture control* will be assessed with: **a**) a computerized dynamic posturography system (Equitest, NeuroCom International, Clackamas, OR) [66]. This system can be used to systematically parse out changes in the visual, vestibular and proprioceptive contributions to postural equilibrium control (see Figure 3). To perform these tests subjects stand quietly on force plates during various combinations of visual and proprioceptive input and are asked to maintain upright stance for 20 s trials. In this study, trials will be performed only with eyes closed and a sway-referenced base (Sensory Organization Test 5) with head erect and while subjects perform dynamic head tilts (forward, backward, or actively moving at 0.33 Hz paced by an audible tone). This condition assesses vestibular control of posture. Assessment time is approximately 10 minutes; **b**) The Functional Mobility Test (see Figure 4) [20]: In each test session subjects will be instructed to “walk without touching any of the obstacles on the course as quickly and as safely as possible without running”. The first half of the 6.0 m × 4.0 m course is set up on a stable hard floor. The second half of the course is set up on a base of 10 cm thick, medium-density foam (Sunmate Foam, Dynamic Systems Inc. Leicester, NC, USA), which makes proprioceptive inputs unreliable during ambulation. The course consists of the following obstacles: 1) a “portal” constructed of two successive 31 cm high Styrofoam blocks placed on concrete surface, with a horizontal foam bar hung from the ceiling between these blocks, the height of which is adjusted to that of the crewmember’s shoulders requiring crewmembers to bend at the waist or lower themselves to avoid hitting the bar hung from the ceiling and balance on a single foot on the stable hard surface while stepping over the barrier; 2) four foam pylons arranged in a “slalom” fashion on the concrete floor, which requires the subject to change heading direction continuously; 3) a 46 cm high Styrofoam block placed on the foam surface which forces the crewmember to balance on one foot on a stable hard surface (floor) while clearing the obstacle and stepping onto the compliant (foam) surface; 4) a gate on the foam surface with edges defined using two foam pylons hung from the ceiling, the width of which is adjusted to the width of the crewmember’s shoulders, so they have to walk between the pylons sideways; and 5) another “portal” (see 1)), but this time placed on the foam surface as the base support.

The crew member once instructed will release the buckle on the chair and continue the course in the following order: 1.) crosses one portal on the stable hard surface; 2) walks on the floor through the vertical “slalom” pylons; 3) turns to the left and presses a button on the wall; 4) turns around and steps over the 46 cm high Styrofoam block; 5) the subject now enters the medium density foam course and presses the button on the wall; 6) the subject turns around and walks through the gate; 7) crosses the second portal on the compliant foam surface; and 8) ends by crossing the first portal. Please note the numbers besides the arrows in Figure 4 denote the order of progression through the course as described above.

This task will be performed ten times. Subjects will be allowed to rest between trials, especially immediately after flight, and all ten trials will be completed within a 10-minute window. To prevent injury from falling, in addition to the medium density foam on the floor, subjects will wear a harness while being monitored by a “spotter”. The primary performance metric will be time to complete the course and number of obstacles hit. Time metrics will be obtained with an optical timing system (Event Timer Pro, Computer Products for Education, Kingston, PA, USA) placed at strategic points on the course to obtain intermediate time measures.

### Vestibular function

**Figure 5:**
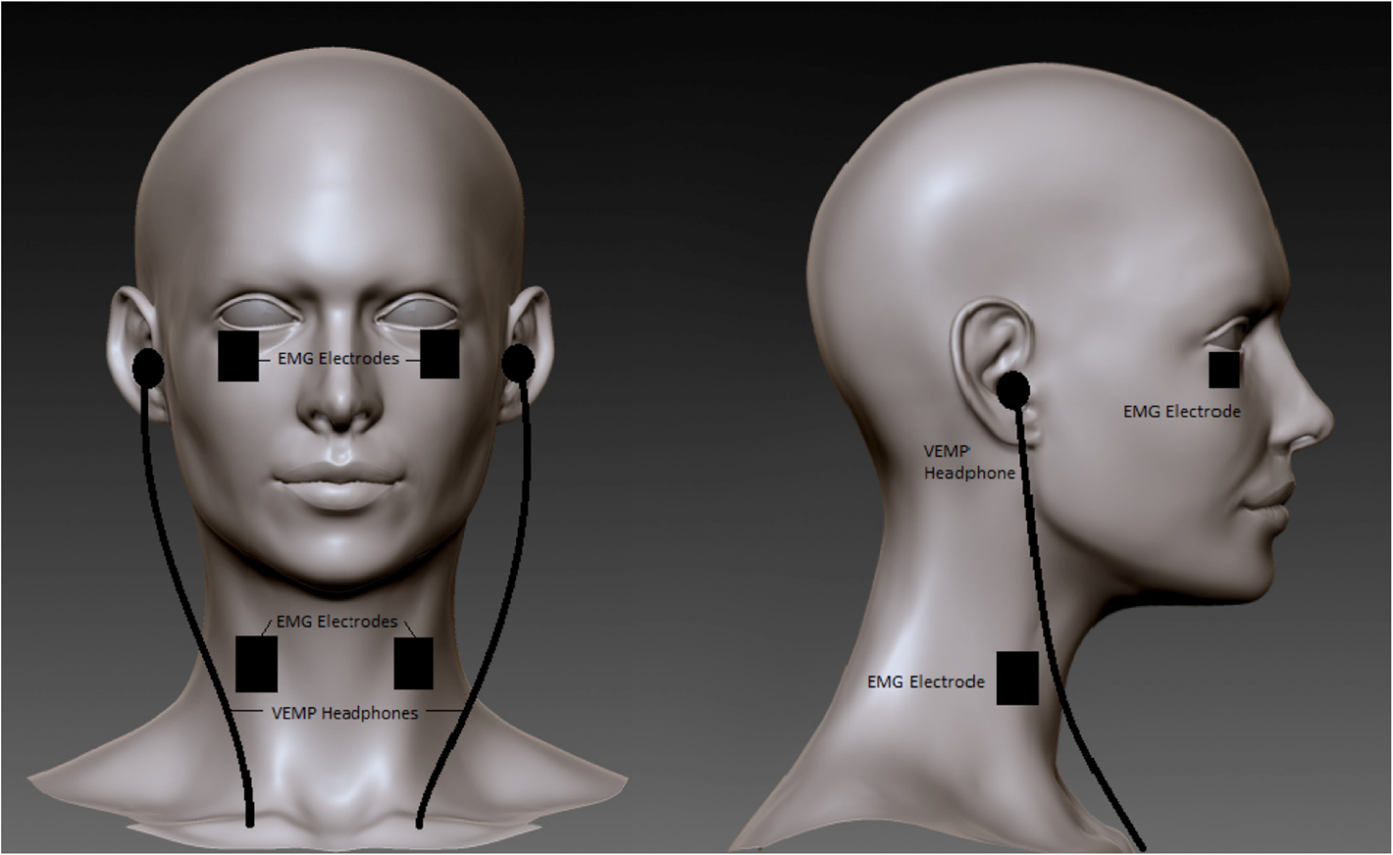
The sound-evoked ocular Vestibular Evoked Myogenic Potential (oVEMP) and colic VEMP (cVEMP).

**Figure 6:**
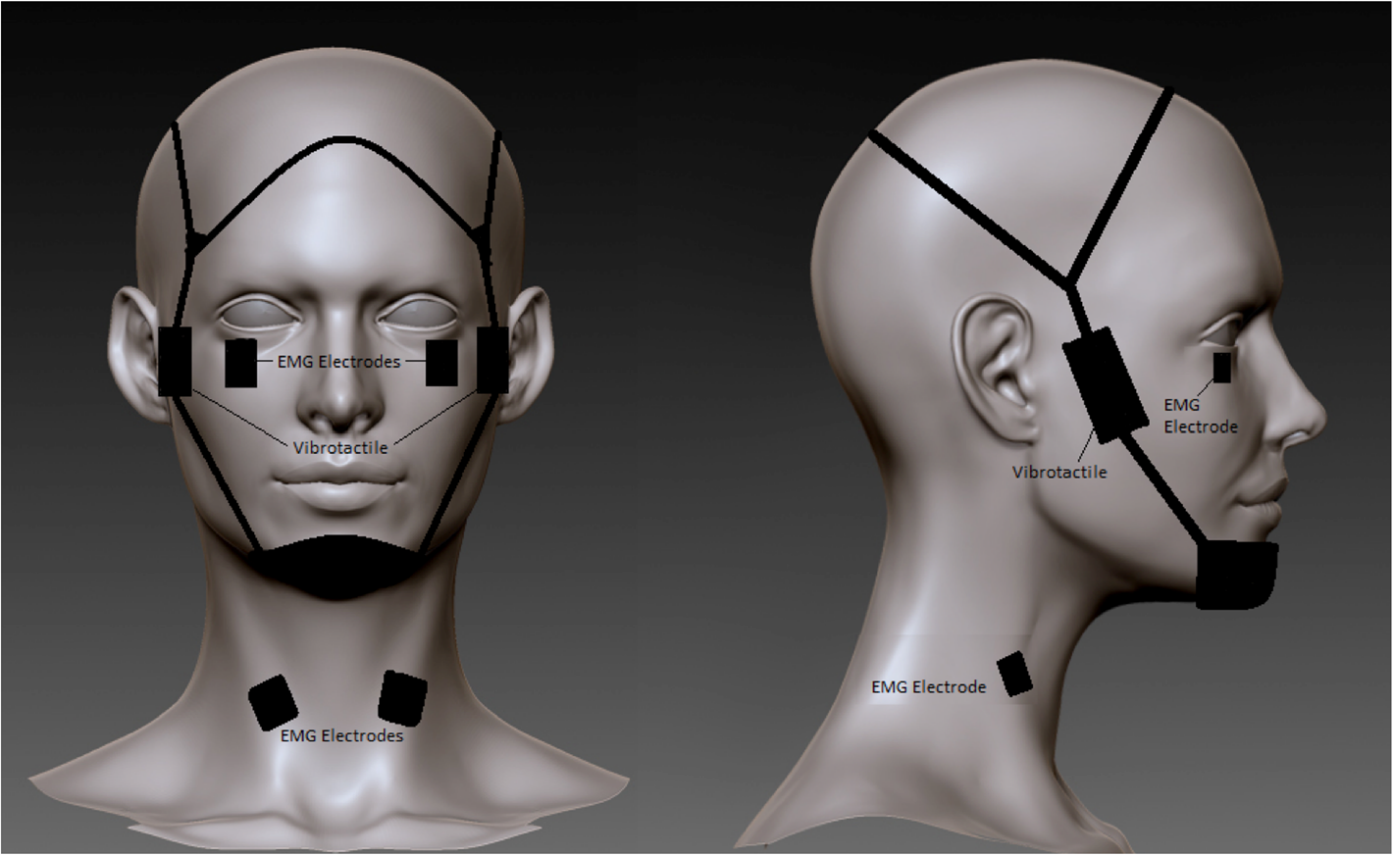
The vibrotactile-evoked ocular Vestibular Evoked Myogenic Potential (oVEMP).

The sound-evoked ocular Vestibular Evoked Myogenic Potential (oVEMP) and colic VEMP (cVEMP) will be elicited by a 500 Hz (8 ms, rate 3 Hz) pure tone of up to 130 dB SPL [67,68] delivered via calibrated headphones as subjects lay supine on a gurney (see Figure 5) [69,70]. Auditory stimuli will be presented monaurally. The vibrotactile-evoked ocular VEMP (oVEMP) will be elicited by a vibrotactile pulse presented at the rate of 1 Hz on the side of the forehead as subjects lay supine on a gurney (see Figure 6) [71].

In a couple of trials, subjects will rotate and raise their heads to contract the sternocleidomastoid muscle for measuring the cVEMP responses. In a separate couple of trials for measuring the oVEMP responses since the response is dependent on gaze direction, subjects will be directed to gaze approximately 25° above straight ahead in semi-darkness.

For the oVEMP electromyograms will be recorded with active bipolar electrodes (Delsys Inc., Boston, MA) on the infraorbital ridge 1 cm below the eyelid with a reference electrode on the chin or sternum or knee cap or the ankle. For the cVEMP electromyograms will also be recorded with active bipolar electrodes from sterocleidomastoid muscles while subjects contract their neck muscles by turning their head by 90 deg and lifting them. The EMG potentials will be amplified, band-pass filtered using a Bagnoli™ Desktop EMG System (Delsys Inc., Boston, MA, USA). This EMG signal is sampled at 10 kHz and the data stimulus onset to 100 ms will be averaged over 100 trial repetition for the auditory VEMP and 24 trial repetitions for the vibrotactile VEMP. The typical oVEMP EMG response is an excitatory potential with first peak occurring at 11–12 ms and second peak at 18 ms. This requires a total recording time of approximately 38 or 29 seconds per trial which includes 5 seconds of 0 dB SPL or no vibrotactile stimulation at the beginning of the protocol. The primary dependent measures consist of the latency and peak-to-peak amplitude from the EMG signals, which will be normalized to EMG levels at the beginning of the protocol.

### Image acquisition

Multi-sequence MRI for astronauts and ground-based control subjects will be performed on the same 3 T Siemens Magnetom verio MRI scanner located at UTMB at Victory Lakes. Multi-sequence MRI for bed rest subjects will be performed on a 3-Tesla Siemens Magnetom skyra syngo MRI scanner located at UTMB at Galveston. The scan protocol for astronauts, bed rest subjects and ground-based controls will be identical. For this study we will use a T1-weighted gradient-echo pulse sequence with the following parameters: 3D T1 axial overlay (TR = 1900 ms, TE = 2.44 ms, flip angle = 9°, FOV = 270 × 270 mm, slice thickness = 0.9 mm, 192 slices; matrix = 288 × 288 voxel size = 0.9375 × 0.9375 × 0.9 = 0.7910 mm^3^, duration = ~4 minutes).

For DTI, we will perform a single shot echo planar sequence (TR = 10100 ms, TE = 95 ms, FOV = 240 × 240 mm, slice thickness = 2.0 mm, matrix = 128 × 128, voxel size = 1.88 × 1.88 × 2.0 mm, 75 contiguous slices. Maximum **b**-value was 1000 s/mm^2^ in 30 non-collinear directions, NEX = 2, and two volumes were acquired without diffusion weighting (**b-**value **=** 0 s/mm^2^)) [72]. Acquisition time is ~11 min. All slices are contiguous.

For “Resting State” functional MRI, we will use a single-shot gradient-echo (GRE) echo planar imaging (EPI) sequence [73] to acquire 240 T2*-weighted BOLD images (TR = 3660 ms, TE = 39 ms, flip angle = 90°, FOV = 240 × 240 mm, slice thickness of 4 mm, 1 mm slice gap, matrix = 94 × 94, voxel size = 2.55 × 2.55 × 5.0 mm, 36 axial slices, duration = ~10 minutes). Participants will be instructed to keep their eyes open, to remain awake, look at a fixation point and to not think about anything in particular. A pulse oximeter will be placed on the participant’s finger to record the cardiac signal, both of which will be regressed out prior to data analyses.

For functional MR imaging we will use a gradient echo T2*-weighted echo-planar imaging (EPI) sequence with BOLD contrast (TR = 3660 ms, TE = 39 ms, flip angle = 90°, FOV = 240 × 240 mm, slice thickness of 4 mm, 1 mm slice gap, matrix = 94 × 94, voxel size = 2.55 × 2.55 × 5.0 mm, 36 axial slices). The EPI images will be collected parallel to the AC-PC line.

### Functional MRI paradigms

Six task-based functional MRI paradigms will be used to assess relationships between (changes in) motor function and brain activation:

*Activation of the vestibular cortex* will be elicited using methods described above without the EMG recordings. We will follow the methods of two recent fMRI investigations of the vestibular cortex using either click-induced VEMP [74,75] or the vibro-tactile stimulation described above. We use approximately 10 blocks of alternating 24- and 20-second periods of on and off stimulation, respectively. They provided stimulation to both the left and the right ear, as well as control auditory stimulation of a lower intensity. Duration of test is ~8 min.

*Activation of the motor and somatosensory regions of the brain* (primary motor cortex, primary sensory cortex, premotor cortex, cerebellum, etc.) will be elicited using stimulus-driven finger tapping (dual task test). Functional MRI will be acquired during the finger tapping under single and dual task conditions while subjects are performing a secondary cognitive task. This dual tasking requirement will allow us to determine whether the predicted compensatory activation (and/or spread of activation due to remapping of the sensorimotor cortex during spaceflight) results in compromised availability of neural resources for dual tasking.

Subjects will view two stimulus boxes on the display screen. They will be instructed to press the matching button when one of the stimulus boxes lights up. Using a pacing stimulus will allow us to keep subjects tapping at the same rate for each of the test sessions, since rate of movement greatly affects the associated patterns of brain activation [76,77]. Stimuli will be presented randomly so that learning will not play a role across test sessions. For some blocks, we will also have subjects perform the tapping task in combination with a secondary, distractor task (cf. [78,79]). This task requires subjects to view another stimulus box centered directly above those just described. This box changes color at a rate of 3 Hz and subjects are instructed to keep track of the number of times that their target color appears. The incidence of the target color is kept quite low (1-3%), forcing the subjects to remain vigilant. The tapping inter-stimulus interval will be 800 ms. The subjects will perform the tapping task and the distractor task in isolation as well as performing both tasks together, in an order that is counterbalanced across participants and test sessions. One example sequence is: 20 seconds control (view static display while not moving), 20 seconds motor, 20 seconds control, 20 seconds dual, 20 seconds control, 20 seconds distractor task (tracking target color), 20 seconds control. This block design will be repeated three times, resulting in nine minutes of image acquisition.

*Brain activation of adaptive learning* will be evaluated using a sensorimotor adaptation task. Sensorimotor adaptation refers to modifying the mapping between sensory and motor space. The task that we selected is one that we have used extensively to study adaptive sensorimotor behavior [80]. Participants move an MRI-compatible joystick to targets presented on a computer display screen, with real-time feedback of the joystick location presented as a cursor on the screen. The adaptive stimulus will be a 45° rotation of the visual feedback display about the central start location. The direction of this visual feedback display rotation will be randomized across testing sessions to overcome any learning effects that may persist across testing session days. Participants will first perform a block of trials under normal visual feedback, followed by several blocks of trials under the rotated feedback condition, and then an additional block of normal feedback to allow us to measure the aftereffects of learning. When we conduct this task in the fMRI environment, each block will have a 20 second visual fixation period at the beginning of the run, and periods of the motor task will alternate with fixation every 40 seconds (block design protocol). The control blocks at the beginning and end of the experiment are important for separating learning related activation from scanner signal drift over the course of the experiment. Moreover, they allow us to identify brain regions that contribute to both motor control and motor learning. This block design will be repeated three times, resulting in ~ thirteen minutes of image acquisition.

*Brain regions actively involved in spatial working memory and mental spatial rotation* are revealed using the spatial working memory task that we have used to study spatial cognitive contributions to sensorimotor adaptation [81-83]. When we conduct this task in the fMRI environment, each block will have a 20 second visual fixation period at the beginning of the run, and periods of the task will alternate with fixation every 36 seconds (block design protocol). The task requires participants to memorize a three-target set (solid circles) in a 500 ms period. Participants are instructed to mentally “connect the dots” of the target set and then mentally rotate the resulting shape 30° clockwise during a 3000 ms retention interval. They are then asked to indicate whether a subsequently presented probe set of circles forms the same configuration as the rotated target shape. The ratio of match to non-match trials will be 70:30. The control task involves visual processing and making a manual response in the absence of working memory demands; that is, the control task does not rely upon spatial working memory or mental rotation. Comparison of the two conditions reveals brain regions actively involved in spatial working memory and mental spatial rotation. This block design will be repeated three times, resulting in ten minutes of image acquisition.

*Brain regions involved in foot movement* will be extracted by having the subject moving their foot while inside the scanner. Subjects are requested to move their right foot by dorsi- and plantar- flexing the ankle joint, paced by a visual stimulus at 1 Hz. Subjects are asked to alternate between 20 seconds of movement and 20 seconds of rest within runs. Duration of test is ~3 min.

#### In flight behavioral assessments

Multiple behavioral outcome measures will be obtained in flight to complement the pre and post-flight testing in astronauts. These tests will include the cube mental rotation test, the dual task test (finger tapping while performing a secondary cognitive task), and the joystick-based sensorimotor adaptation test that we will conduct during fMRI scanning pre- and post flight (see under 'behavioral assessment' and 'functional MRI paradigms).

The mental rotation test will be performed under two postural conditions; one is with the subject in a 'seated' posture using a harness and foot loop with the feet flat on the floor. The dual task test and sensorimotor adaptation test will also be performed under this configuration. Then the mental rotation test will be performed again with the subject floating unconstrained with the exception of a waist tether.

## Discussion

This study is the first interdisciplinary assessment of the effects of space flight on brain structure and function, cognition and behavioral performance changes in astronauts. Because of the longitudinal design of the study with implementation of pre-launch assessments, the astronauts are able to serve as their own controls. By using a well-established spaceflight analog (head down tilt bed rest) we will be able to gain insight into the underlying neural mechanisms and operational risks of spaceflight-induced changes in behavior. Inclusion of an additional group of ground-based control subjects will aid in estimating the stability and reliability of our behavioral and MRI measures over time.

The longitudinal design of the study allows for determining how brain neuro-structural changes impact functional performance. This understanding could aid in the design of targeted countermeasures to mitigate the negative effects of long-duration spaceflight. Because we will obtain multiple post flight measures, we will be able to study if the hypothesized return to normative behavioral function following recovery from prolonged spaceflight is associated with a restitution of brain structure and function or instead is supported by substitution with compensatory brain processes.

Nevertheless, we are aware that our study has some limitations that need to be addressed. This study comprises three single group-longitudinal designs, rather than a group-by-time design in which both control groups would be testing along the same time points as the astronauts. The latter design would be better suited for discerning the effects of microgravity from other factors encountered in space, unraveling group-by-time interactions, and regressing out the effects of shifts in measurement precision of equipment over time. Such a design however is not feasible because there is not a one-to-one temporal match between the effects of spaceflight and the effects of prolonged bed rest on sensorimotor control. However, if we identify systematic changes over time in the ground-based control group, the astronauts’ and bed rest participants’ data can be de-trended to account for drifts. Moreover, if any of our tests show poor test-retest reliability, we can omit and/or replace them for the crewmember and bed rest tests.

The number of astronauts to be included in the current study is aimed at thirteen. Proper sample size calculation revealed this number should be sufficient to accurately estimate behavioral changes from pre- to post space flight. Nevertheless, the relatively small sample size hinders inclusion of multiple confounders in statistical models, and makes it difficult to detect small changes. However, considering the absolute small number of astronauts that participate in long duration space flight, the proposed sample size is reasonable in terms of power and feasibility.

The major strength of this study is its unique nature. No prior studies have examined the effects of spaceflight on the extent, longevity and neural bases of sensorimotor and cognitive performance in human subjects. This study integrates high priority research topics in NASA’s Human Research Program, which seeks to estimate the risk of impaired control of a spacecraft, associated systems, and immediate vehicle egress due to vestibular and/or sensorimotor alterations that are associated with spaceflight. Moreover, the current study addresses the health and performance of crewmembers in flight and post-flight, which is important from a well-being perspective as well as in light of success of space flight missions. The results should also prove informative regarding the adaptive capacity of brain and behavior.

## Abbreviations

AC-PC: Anterior commissure posterior commissure; BOLD: Blood oxygenation level-dependent; CNS: Central nervous system; cVEMP: Colic vestibular evoked myogenic potential; DTI: Diffusion tensor imaging; EMG: ElectroMyoGram; EPI: Echo planar imaging; fMRI: functional magnetic resonance imaging; FMT: Functional mobility test; GRE: GRadient echo; ISS: International space station; MRI: Magnetic resonance imaging; NASA: National aeronautics and space Administration; NEX: Number of EXcitations; oVEMP: ocular vestibular evoked myogenic potential; RFT: Rod and frame test; TE: Echo time; TR: Repetition time; UTMB: University of texas medical branch; VEMP: Vestibular evoked myogenic potential; WAIS-R: Wechsler adult intelligence scale-revised.

## Competing interests

The authors declare that they have no competing interests.

## Authors’ contributions

RDS, PRL, SJW, IK, JJB, and APM are accountable for the conception and the design of the study. Data collection is lead and executed by YEDD, and APM. VK and BE are responsible for the data analysis. All authors have participated in the draft and the revision of the manuscript, and have approved its final version.

## Pre-publication history

The pre-publication history for this paper can be accessed here:

http://www.biomedcentral.com/1471-2377/13/205/prepub
